# Perioperative antibiotic prophylaxis in robotic-assisted ventral hernia repair: a propensity score-matched analysis

**DOI:** 10.1007/s10029-026-03805-5

**Published:** 2026-07-31

**Authors:** Fadl Alfarawan, Harmeet singh Sodhi, Liv Faulhaber, Ahmed Al-Mawsheki, Gernot M. Kaiser, Maximilian Bockhorn

**Affiliations:** 1 Fakultät für Gesundheitswissenschaften, Carl von Ossietzky Universität Oldenburg, Ammerländer Heerstraße 114-118, Oldenburg, 26129 Germany; 2 Department for General – and Visceral Surgery, University Hospital Oldenburg, Klinikum Oldenburg AöR, Rahel-Straus-Straße 10, 26133 Oldenburg, Germany

**Keywords:** Robotic ventral hernia repair, Antibiotic prophylaxis, Surgical site infection, Surgical site occurrence, Propensity score matching, Antimicrobial stewardship

## Abstract

**Background:**

Routine antibiotic prophylaxis is widely used in the treatment of ventral hernias, although high-quality evidence supporting its necessity in modern minimally invasive procedures with extraperitoneal mesh placement is limited. Growing concerns regarding antimicrobial stewardship warrant a reassessment of this practice.

**Methods:**

We conducted an ambidirectional cohort study with a historical (retrospective) control group of consecutive adult patients who underwent an rVHR (eTEP, TAPP, or eTEP/TAR) between June 2023 and December 2025 at a single tertiary center. Patients treated before January 2025 received single-shot prophylaxis as the institutional standard and were identified retrospectively from a prospectively maintained surgical database (historical control cohort); from January 2025 onwards, following a revised institutional protocol omitting routine prophylaxis, patients were enrolled prospectively and followed systematically through 90 days postoperatively (prospective intervention cohort). Patients were stratified based on the administration of perioperative single-shot antibiotic prophylaxis. To address potential selection bias, 1:1 nearest-neighbor propensity score matching (PSM) was performed with a caliper of 0.1 of the logit of the propensity score. The matching variables included a range of sociodemographic and health-related characteristics, including age, hernia category, BMI, ASA classification, diabetes status, smoking status, and the size of the hernia defect. The balance of the groups was evaluated using standardized mean differences (SMD, threshold < 0.10). The primary endpoints were defined as SSO and SSI at 30 and 90 days, while the secondary endpoints included LOS as well as univariate and multivariate odds ratios (OR). In addition, prespecified subgroup analyses were performed with respect to surgical technique, BMI class, smoking status, diabetes mellitus, anticoagulation, and wound class.

**Result:**

Of the 260 patients included in the study, 258 met the inclusion criteria, of whom 174 (67.4%) received antibiotic prophylaxis and 84 (32.6%) did not. PSM yielded 78 well-balanced pairs (all post-match SMDs ≤ 0.13, propensity score SMD 0.007). The rate of 30-day SSOs did not differ significantly between the matched groups (12.8% with prophylaxis vs. 15.4% without, McNemar *p* = 0.823), nor did the rate of 90-day SSOs (5.1% vs. 3.8%, *p* = 1.000). 30-day SSIs were rare and comparable between the groups (2.6% vs. 1.3%, *p* = 1.000); no SSIs occurred after 90 days. The median length of stay (LOS) was two days in both groups (Wilcoxon *p* = 0.507). Multivariable logistic regression confirmed that there was no association between prophylaxis and 30-day SSOs (adjusted odds ratio [aOR] 1.28; 95% CI 0.58–2.82; *p* = 0.543) or SSIs (aOR 1.92; 95% CI 0.18–20.31; *p* = 0.588). In the subgroup analyses, no stratum could be identified in which prophylaxis significantly reduced SSOs. There was only a non-significant trend toward higher SSO rates in obese patients (Grade II/III) who received prophylaxis (30.4% vs. 7.1%; OR 4.02; 95% CI 0.67–24.22).

**Conclusion:**

In this propensity-score-matched cohort, the omission of perioperative antibiotic prophylaxis following rVHR is not associated with an increase in SSOs, SSIs, or LOS. In conjunction with the very low overall SSI rate (approx. 2%), these findings prompt a necessary shift toward a more differentiated approach to routine antibiotics in this setting. We emphasize, however, that this conclusion must not be extrapolated to laparoscopic intraperitoneal onlay mesh (IPOM) or to open repair, where the contamination profile and wound-healing risk differ fundamentally.

## Introduction

The repair of ventral hernias represents one of the most commonly performed procedures in general and visceral surgery worldwide. In Germany alone, an estimated 122,000 procedures are performed each year [1, 2]. The clinical and socioeconomic relevance is further underscored by the fact that the incidence of incisional hernia formation reaches 22.4% within three years following abdominal surgery [3]. The significant reduction in wound-related morbidity compared to open repair is attributable to the widespread adoption of minimally invasive techniques and, more recently, robot-assisted approaches [4, 5]. As a result of this development, retromuscular mesh placement using the Enhanced-View Totally Extraperitoneal (eTEP) technique, the transabdominal preperitoneal (TAPP) approach, and the Transversus Abdominis Release (TAR) have established themselves as gold standards for incisional ventral hernias [4–6].

Routine perioperative antibiotic prophylaxis is a nearly universal component of clinical protocols for hernia repair and is recommended by current guidelines from professional societies, including the World Society of Emergency Surgery and the European Hernia Society [7, 8]. The underlying data originate from open hernia repair and demonstrate that the rate of Surgical Site Infections (SSI) can be reduced by a single intravenous antibiotic dose administered within 60 min prior to the skin incision [9].

However, the current risk profile of robot-assisted ventral hernia repair (rVHR) differs fundamentally from historical open cohorts: typical SSI rates in modern series are below 5%, mesh placement is predominantly retromuscular rather than intraperitoneal, and the extent of intraoperative contamination is minimal [10–12]. Furthermore, growing concerns regarding antimicrobial stewardship, as well as documented side effects of even short-term prophylaxis, such as disruption of the gastrointestinal microbiome, selection of resistant pathogens, and rare but severe hypersensitivity reactions, have led to increasing scrutiny of the routine administration of prophylaxis in clean elective surgery [13, 14]. Currently, there is no high-quality evidence available that would allow for a targeted investigation of the necessity of prophylaxis in rVHR. Furthermore, significant interinstitutional variations are evident in documented clinical practice [15].

We hypothesized that in modern rVHR with primarily extraperitoneal mesh placement, the omission of perioperative antibiotic prophylaxis is not associated with an increased rate of Surgical Site Occurrences (SSOs) or SSIs at 30 or 90 days. The primary objective of this study was to compare 30- and 90-day SSO/SSI rates between patients with and without single-shot prophylaxis using propensity score matching to address potential selection bias. Secondary objectives included evaluating length of hospital stay, estimating adjusted odds ratios using multivariate logistic regression, and identifying subgroups (surgical technique, BMI class, smoking status, diabetes mellitus, anticoagulation, wound class) in which prophylaxis might offer differentiated benefits.

## Materials and methods

### Study design and population

We conducted an ambidirectional cohort study, comprising a prospectively enrolled intervention cohort and a historical retrospective control cohort, of all consecutive adult patients (aged 18 years and older) who underwent rVHR at our institution between June 2023 and December 2025. Until December 2024, single-shot perioperative antibiotic prophylaxis was the institutional standard for elective rVHR; these patients constitute the historical (retrospective) control cohort and were identified from a prospectively maintained surgical database. Following a revised institutional protocol implemented in January 2025 that omitted routine prophylaxis, all subsequent rVHR patients were enrolled prospectively and followed systematically through 90 days postoperatively (prospective intervention cohort). This study was conducted in accordance with the STROBE guidelines for reporting observational studies [16]. Data were extracted from a prospectively maintained surgical database and cross-validated against electronic medical records by an independent investigator. Approval from the Institutional Review Board was obtained. As the omission of antibiotic prophylaxis from January 2025 onwards reflected a routine institutional protocol change rather than a research intervention, and given the use of de-identified data, the institutional review board waived the requirement for individual informed consent.

The inclusion criteria are defined as follows: adult patients who underwent elective rVHR, with the procedure performed using eTEP, TAPP, or eTEP/TAR with mesh augmentation.

The exclusion criteria are outlined below. Among surgical interventions, emergency procedures were performed for incarcerated or strangulated hernias with bowel resection. The data regarding exposure to antibiotic prophylaxis were insufficient (*n* = 2). In addition, simultaneous non-hernia-related procedures, such as cholecystectomy, were performed. After applying these criteria, 258 of 260 consecutive cases remained in the analytical dataset.

### Antibiotic prophylaxis protocol

During the study period, the in-house protocol recommended a single intravenous dose (single-shot) of 2 g cefazolin (3 g for patients weighing > 120 kg) within a 60-minute window prior to the skin incision. In patients with a documented β-lactam allergy, this was substituted with 600 mg of clindamycin. The decision regarding prophylaxis was made by the operating surgeon and documented in the anesthesia record. Patients without documented antibiotic administration formed the non-prophylaxis cohort; patients who received a prophylactic dose formed the prophylaxis cohort. Therapeutic postoperative antibiotic administration was recorded separately and was not considered prophylaxis. These administrations were indicated in cases of manifest or highly suspected infection.

### Variables and outcomes

Preoperative variables included demographics (age, sex), BMI, the ASA classification, the hernia category (primary vs. recurrent), the defect size in cm² (measured intraoperatively according to the European Hernia Society classification [17]), comorbidities (diabetes mellitus, COPD, pre-existing cardiac conditions, liver failure), smoking status, anticoagulation, and prior abdominal surgeries. Intraoperative variables included the surgical technique (eTEP, TAPP, eTEP/TAR), mesh size, console and skin-to-skin suture times, conversion rates, intraoperative complications, placement of drains, and intraoperative transfusions.

The primary endpoint was defined as the composite SSO outcome at 30 days, as described by Petro and Novitsky [18]. The present classification includes superficial SSI (surgical site infection), deep or organ/space infection, seroma, hematoma, wound dehiscence, enterocutaneous fistula, or any wound complication requiring intervention. SSOs were further specified as SSIs (if pathogens were detected) or as SSOs requiring procedural intervention (SSOPI) if drainage, bedside wound debridement, or revision surgery was necessary. Secondary endpoints included SSOs/SSIs at 90 days, length of hospital stay (LOS), rehospitalizations within 30 days, postoperative ileus, and the Clavien-Dindo grade [19].

### Surgical technique

Standardized rVHR was performed using the da Vinci X system (Intuitive Surgical, Sunnyvale, CA). Using the eTEP technique, the retromuscular space was dissected bilaterally with a crossover at the linea alba, a defect closure was created, the posterior rectus sheath was re-adapted, and a polypropylene mesh (Bard^®^ Soft Mesh) was placed in the retromuscular compartment. TAR was indicated if the transverse defect width exceeded 8–10 cm or if sufficient retromuscular dissection could not otherwise be achieved [20, 21]. The TAPP technique was used for primary umbilical and epigastric hernias with rectus diastasis, whereby the mesh (Bard^®^ Soft Mesh) was unfolded in the preperitoneal layer after defect closure and linea alba approximation. Closure of the hernia defect was performed in all cases using slow-absorbing, self-fixing suture material (STRATAFIX^®^ 0). The mesh was fixed either by its self-adhesive properties or by absorbable single-knot sutures (Vicryl 3 − 0) [22].

### Selection of the surgical technique

The robotic technique was selected according to a standardized institutional algorithm based on hernia category and defect characteristics (Fig. 3). For primary hernias, a ventral transabdominal preperitoneal repair (vTAPP) was used for defects of 1–7 cm and for rectus diastasis or multiple midline defects, whereas defects larger than 7 cm were treated by eTEP with transversus abdominis release (TAR) added as required. For incisional hernias, vTAPP was used for defects ≤ 5 cm without a prior laparotomy, eTEP for defects ≤ 5 cm with a prior laparotomy, and eTEP with TAR for defects larger than 5 cm. Special presentations were managed individually: lateral hernias (L1–L4) by vTAPP and parastomal hernias by eTEP with TAR. The mesh plane followed directly from the technique chosen (preperitoneal for vTAPP; submuscular/retromuscular for eTEP and eTEP/TAR).

### Statistical analysis

Continuous variables are presented as mean ± standard deviation (SD) or median with interquartile range (IQR), depending on their distribution. Categorical variables are presented as absolute frequency (n) and percentage (%). For univariate comparisons, the chi-square test or Fisher’s exact test was used for categorical data, and the t-test or Mann-Whitney U test was used for continuous data. To address baseline imbalances and confounding by indication, a 1:1 nearest-neighbor propensity score matching (PSM) without replacement was performed with a caliper of 0.1 on the logit propensity score scale. Propensity scores were generated using a multivariate logistic regression model. Prophylaxis (yes/no) served as the dependent variable, while the following prespecified confounders served as covariates: age, hernia category (primary vs. recurrent), BMI, ASA score, diabetes mellitus, smoking status, and defect size (cm²). Balance was evaluated using the standardized mean difference (SMD). Values of < 0.10 thus indicate a negligible imbalance, while values of < 0.20 are considered acceptable [23].

In the corresponding cohort, the McNemar test was used to compare paired proportions for binary endpoints (SSO and SSI at 30 and 90 days). In addition, the Wilcoxon signed-rank test was used for paired LOS. In the overall cohort, univariate odds ratios (OR) were calculated using 2 × 2 contingency tables with Haldane-Anscombe correction. Multivariate logistic regression provided adjusted odds ratios (aOR) for SSOs and SSIs at 30 and 90 days, adjusted for the seven matching covariates. For 30-day SSOs, prespecified subgroup analyses were performed by stratifying according to surgical technique (eTEP, TAPP, eTEP/TAR), wound class (clean vs. clean-contaminated), BMI category (normal weight, overweight, obesity grade I, obesity grade II/III), smoking status, anticoagulation, and diabetes. Three further pre-specified subgroups were analysed: mesh position (submuscular/retromuscular for eTEP and eTEP/TAR vs. preperitoneal for vTAPP), an infection-risk composite (diabetes mellitus, age ≥ 75 years, hepatic insufficiency, or significant cardiac comorbidity), and hernia category (primary vs. incisional/recurrent). The ORs with 95% CI were calculated in each stratum using the Fisher’s exact test. The tests were conducted as two-sided tests with a significance level of α = 0.05. The analyses were performed in Python (v3.11; pandas, scipy, statsmodels) and subsequently verified by an independent biostatistician using R (v4.3).

## Results

### Cohort characteristics

**Fig. 1:**
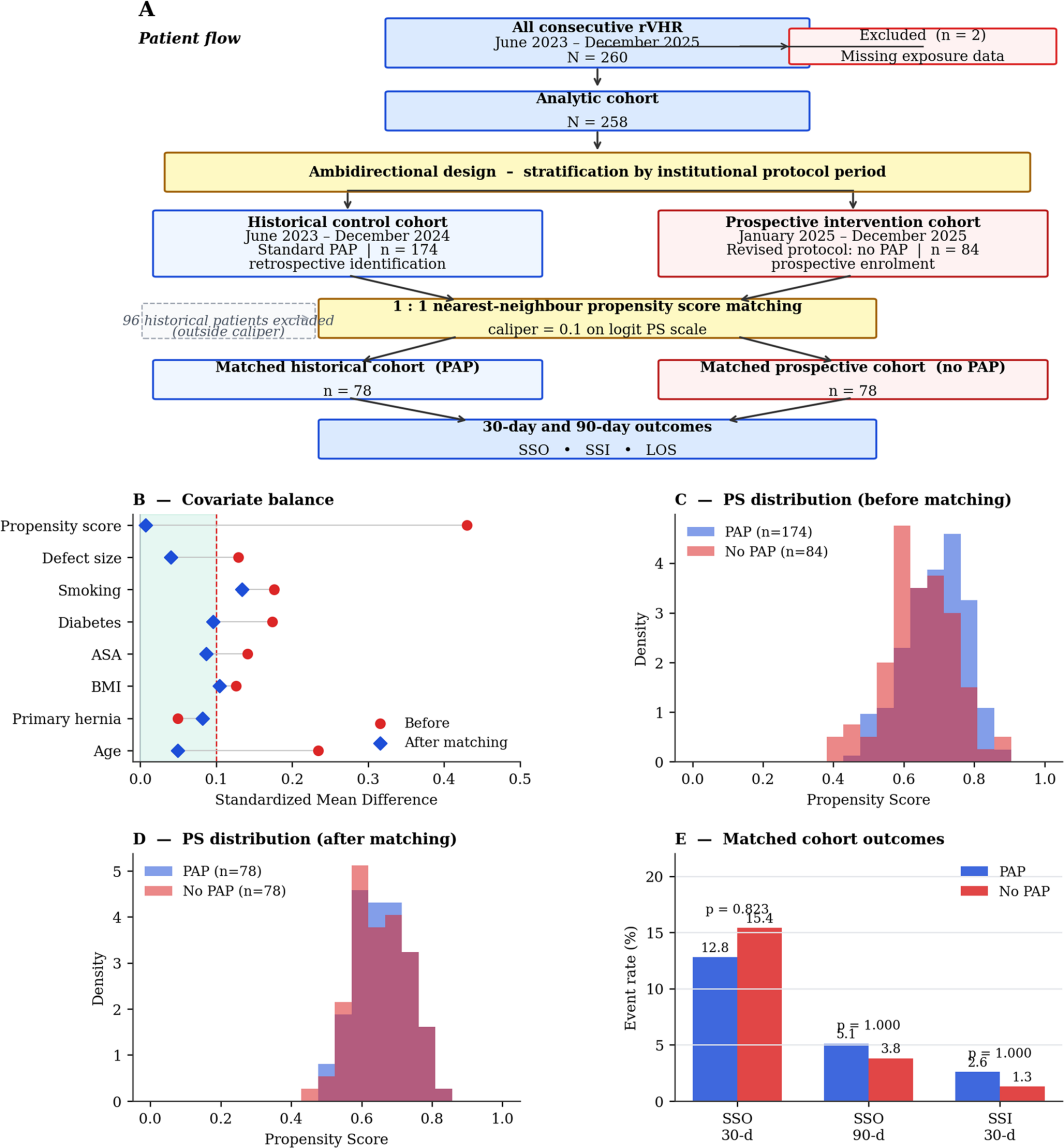
Study design and propensity score diagnostics. (**A**) Patient flow: historical control cohort (June 2023–December 2024, perioperative antibiotic prophylaxis as institutional standard, retrospective identification) and prospective intervention cohort (January 2025–December 2025, no prophylaxis under revised protocol, prospective enrolment). 1:1 nearest-neighbour PSM with caliper 0.1 on logit PS scale yielded 78 matched pairs (96 historical patients excluded, outside caliper). (**B**) Love plot of standardized mean differences (SMD) before (circles) and after (diamonds) matching; dashed line = SMD threshold of 0.10. (**C**, **D**) Propensity score distributions before and after matching. (**E**) Event rates in matched cohort with McNemar p-values. PAP = perioperative antibiotic prophylaxis; SSO = surgical site occurrence; SSI = surgical site infection; LOS = length of stay

The historical control cohort (June 2023 to December 2024) comprised 174 patients who received perioperative antibiotic prophylaxis, while the prospective intervention cohort (January 2025 to December 2025) included 84 consecutive patients managed without prophylaxis under the revised institutional protocol. Overall, 260 consecutive patients underwent rVHR during the study period. After excluding two patients for whom no prophylaxis data were available, 258 patients remained in the analytical cohort. Of the study participants, 174 (67.4%) received perioperative antibiotic prophylaxis, while 84 (32.6%) did not. The unmatched cohort had a mean age of 56.6 ± 14.5 years, a mean BMI of 30.6 ± 5.2 kg/m², and 51.9% were female. The eTEP procedure was performed in 116 patients (45.0%), the TAPP procedure in 98 patients (38.0%), and the eTEP/TAR procedure in 44 patients (17.0%). The hernia categories consisted of 33% primary ventral hernias and 67% incisional or recurrent hernias (Fig. 1).

Prior to matching, there was a substantial imbalance in baseline characteristics, particularly regarding age (SMD 0.234), ASA score (SMD 0.141), diabetes (SMD 0.174), smoking status (SMD 0.176), and defect size (SMD 0.129). The propensity score showed a significant difference between the groups prior to matching (SMD 0.430), reflecting the selective administration of prophylaxis in patients with more complex hernias. The detailed baseline characteristics before and after PSM are presented in Table 1.

### Propensity score matching

PSM (caliper 0.1) resulted in 78 matched pairs (78 with prophylaxis, 78 without prophylaxis), with 96 prophylaxis patients excluded due to a lack of an adequate match within the caliper. The post-match balance showed excellent values for the propensity score (SMD 0.007) and good to acceptable values for all matching covariates (all SMDs ≤ 0.134; five out of seven < 0.10). The post-match SMDs were as follows: age 0.049, primary hernia 0.082, BMI 0.104, ASA 0.087, diabetes 0.096, smoking status 0.134, and defect size 0.040 (Table 1; Fig. 1).

**Table 1:** Baseline characteristics before and after propensity score matching

Variable	Unmatched cohort (*n* = 258)			Matched cohort (78 pairs)		
	AB+	AB−	SMD	AB+	AB−	SMD
Age, years (mean)	55.5	58.7	0.234	58.7	58.0	0.049
Primary hernia, %	32.2	34.5	0.049	29.5	33.3	0.082
BMI, kg/m²	30.8	30.1	0.126	29.4	30.0	0.104
ASA score (mean)	2.24	2.32	0.141	2.23	2.28	0.087
Diabetes mellitus, %	13.8	8.3	0.174	9.0	6.4	0.096
Active smoking, %	26.4	34.5	0.176	38.5	32.1	0.134
Defect size, cm²	55.6	46.7	0.129	47.5	45.2	0.040
Propensity score	0.69	0.65	0.430	0.65	0.65	0.007

*AB +*  received antibiotic prophylaxis, *AB−* no antibiotic prophylaxis, SMD standardized mean difference. Threshold for adequate balance: SMD < 0.10

### Intraoperative characteristics

In the matched cohort, the distribution of surgical techniques between the groups was comparable: eTEP in 56.4% vs. 35.9%, TAPP in 26.9% vs. 51.3%, and eTEP/TAR in 16.7% vs. 12.8% in the prophylaxis and non-prophylaxis groups, respectively. The performance of a TAR was less common in the non-prophylaxis group, which is consistent with the selective use of prophylaxis in more complex reconstructions. The median operative time (incision to suture) was 95 min (IQR 75–135) in the prophylaxis group and 90 min (IQR 70–120) in the control group (*p* = 0.34). Intraoperative complications occurred in 2.6% of the matched pairs in both groups; there were no conversions to open or laparoscopic surgery. No significant differences were observed regarding mesh size, the placement of drains, or estimated blood loss 

### Primary outcome: SSO at 30 and 90 days

In the group that underwent a matching procedure, the 30-day SSO rate was 12.8% (10/78) in the prophylaxis group compared to 15.4% (12/78) in the non-prophylaxis group (McNemar *p* = 0.823). After a 90-day observation period, SSO rates were documented at 5.1% (4/78) and 3.8% (3/78), respectively (*p* = 1.000). The most commonly diagnosed SSO was seroma, followed by hematoma. Hematomas requiring intervention (SSOPI) occurred in one of 78 pairs in each group. There are no observations suggesting that mesh-associated complications or enterocutaneous fistulas occurred after 30 or 90 days.

### Secondary outcomes: SSI and length of stay

30-day SSIs were a rare complication and showed no significant differences between the matched groups: 2.6% (2/78) with prophylaxis versus 1.3% (1/78) without prophylaxis (*p* = 1.000). The present study found that all cases examined were superficial cellulitis, which could be successfully treated with oral antibiotics. In no case was reoperation or mesh removal necessary. After a 90-day follow-up period, no SSI events were recorded in either group. The median length of stay (LOS) was two days in both groups (prophylaxis group: IQR 2–3; non-prophylaxis group: IQR 2–2; Wilcoxon *p* = 0.507).

Rehospitalizations within a 30-day period were observed in 5.1% (4/78) of the prophylaxis pairs and 3.8% (3/78) of the control pairs (*p* = 1.000) (Table 2).

**Table 2:** 30- and 90-day outcomes in the propensity score matched cohort (78 pairs)

Outcome	AB+ (*n* = 78)	AB− (*n* = 78)	Test	*p*-value
SSO 30-day, n (%)	10 (12.8%)	12 (15.4%)	McNemar	0.823
SSO 90-day, n (%)	4 (5.1%)	3 (3.8%)	McNemar	1.000
SSI 30-day, n (%)	2 (2.6%)	1 (1.3%)	McNemar	1.000
SSI 90-day, n (%)	0 (0%)	0 (0%)	—	—
LOS, days (median, IQR)	2 (2–3)	2 (2–2)	Wilcoxon	0.507
30-day readmission, n (%)	4 (5.1%)	3 (3.8%)	McNemar	1.000
Postoperative ileus, n (%)	3 (3.8%)	2 (2.6%)	McNemar	1.000

*AB +*  with antibiotic prophylaxis, *AB−* without, SSO  surgical site occurrence (composite of SSI, seroma, haematoma, wound dehiscence, enterocutaneous fistula); *SSI*  surgical site infection, *LOS* length of hospital stay. Discordant pairs for SSO 30-day: b = 9, d = 11

### Univariable and multivariable logistic regression

In the complete unmatched cohort, univariate analysis showed no significant association between prophylaxis and 30-day SSOs (OR 1.27; 95% CI 0.62–2.60; *p* = 0.625) or 90-day SSOs (OR 1.20; 95% CI 0.33–4.27; *p* = 1.000), nor with 30-day SSIs (OR 1.47; 95% CI 0.23–9.50; *p* = 1.000). Multivariate logistic regression (adjusted for the seven matching covariates) yielded nearly identical estimates: The aOR for 30-day SSOs was 1.28 (95% CI 0.58–2.82; *p* = 0.543), for 90-day SSOs 1.49 (95% CI 0.35–6.37; *p* = 0.594), and for 30-day SSIs 1.92 (95% CI 0.18–20.31; *p* = 0.588). The nearly identical univariate and adjusted estimates indicate low residual confounding due to the variables included in the model (Table 3).

**Table 3:** Univariable and multivariable odds ratios for the effect of antibiotic prophylaxis (full cohort, *n* = 258)

Outcome	Events AB+	Events AB−	OR (95% CI)	*p*-value
Univariable				
SSO 30-day	31/174	12/84	1.27 (0.62–2.60)	0.625
SSO 90-day	8/173	3/84	1.20 (0.33–4.27)	1.000
SSI 30-day	4/174	1/84	1.47 (0.23–9.50)	1.000
Multivariable*				
SSO 30-day	—	—	1.28 (0.58–2.82)	0.543
SSO 90-day	—	—	1.49 (0.35–6.37)	0.594
SSI 30-day	—	—	1.92 (0.18–20.31)	0.588

*Adjusted for age, hernia category, BMI, ASA, diabetes, smoking, defect size. OR > 1 favors no prophylaxis (i.e., higher event rate with prophylaxis); OR < 1 favors prophylaxis. All confidence intervals include unity

### Subgroup analyses

**Fig. 2:**
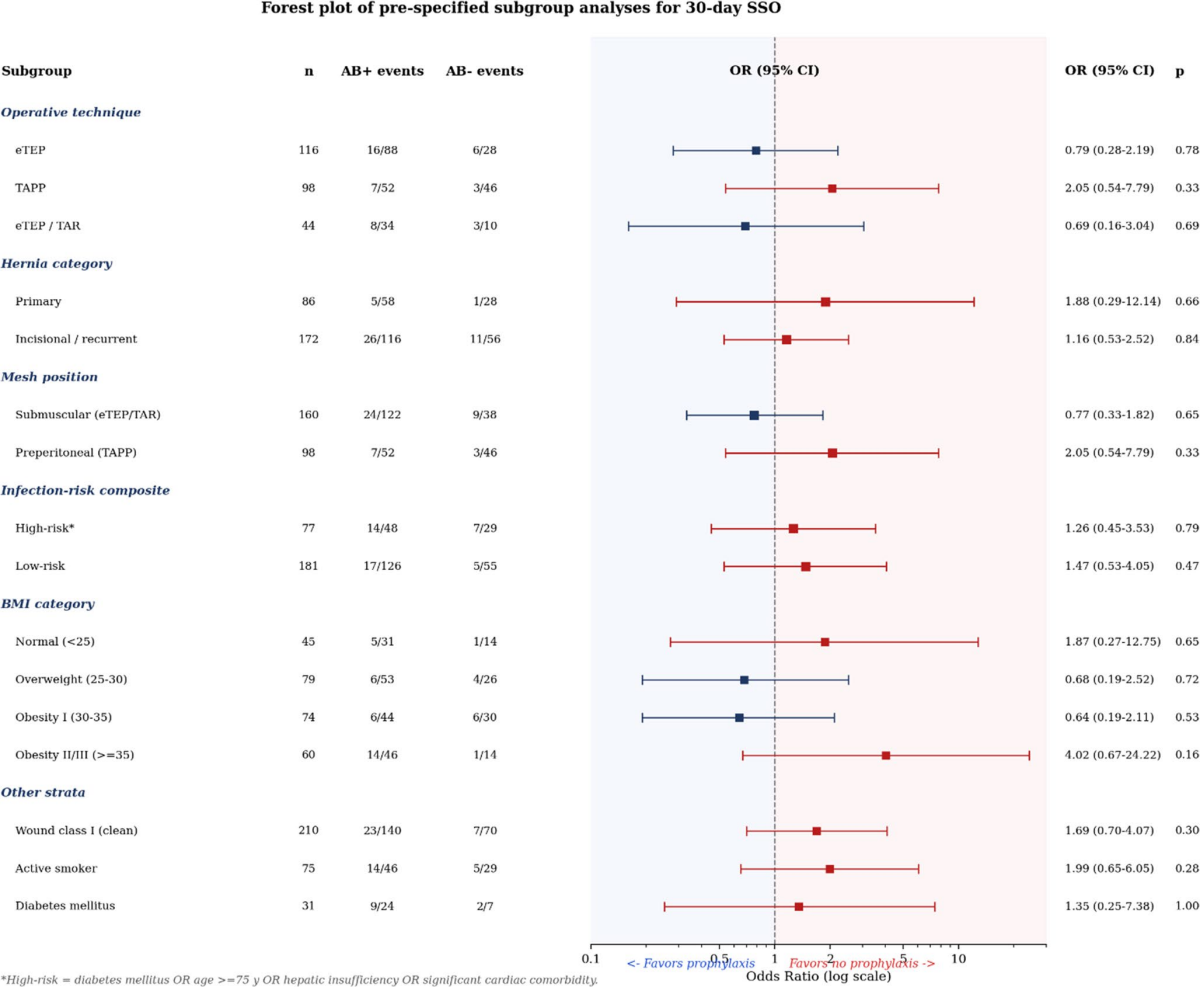
OR > 1 indicates higher SSO rate in patients receiving antibiotic prophylaxis (i.e., favors no prophylaxis). All confidence intervals include unity. eTEP = enhanced-view totally extraperitoneal; TAPP = transabdominal preperitoneal; TAR = transversus abdominis release. *High-risk = diabetes mellitus OR age ≥ 75 years OR hepatic insufficiency OR significant cardiac comorbidity. Mesh position: submuscular/retromuscular (eTEP,* eTEP/TAR) vs. preperitoneal (vTAPP)*

The prespecified subgroup analyses for 30-day SSOs are presented in Table 4. No statistically significant benefit of prophylaxis was observed in any of the analyzed subgroups. The point estimates showed some heterogeneity between the surgical techniques: The results of the present study demonstrate that in eTEP (*n* = 116), prophylaxis was associated with an OR of 0.79 (95% CI 0.28–2.19). For TAPP (*n* = 98), an OR of 2.05 (0.54–7.79) was observed, and for eTEP/TAR (*n* = 44), an OR of 0.69 (0.16–3.04). Classification of wound class using the OR method yielded an odds ratio of 1.69 (0.70–4.07) for clean wounds and an odds ratio of 0.55 (0.15–2.04) for clean-contaminated wounds, although neither result was statistically significant (Fig. 2).

**Fig. 3:**
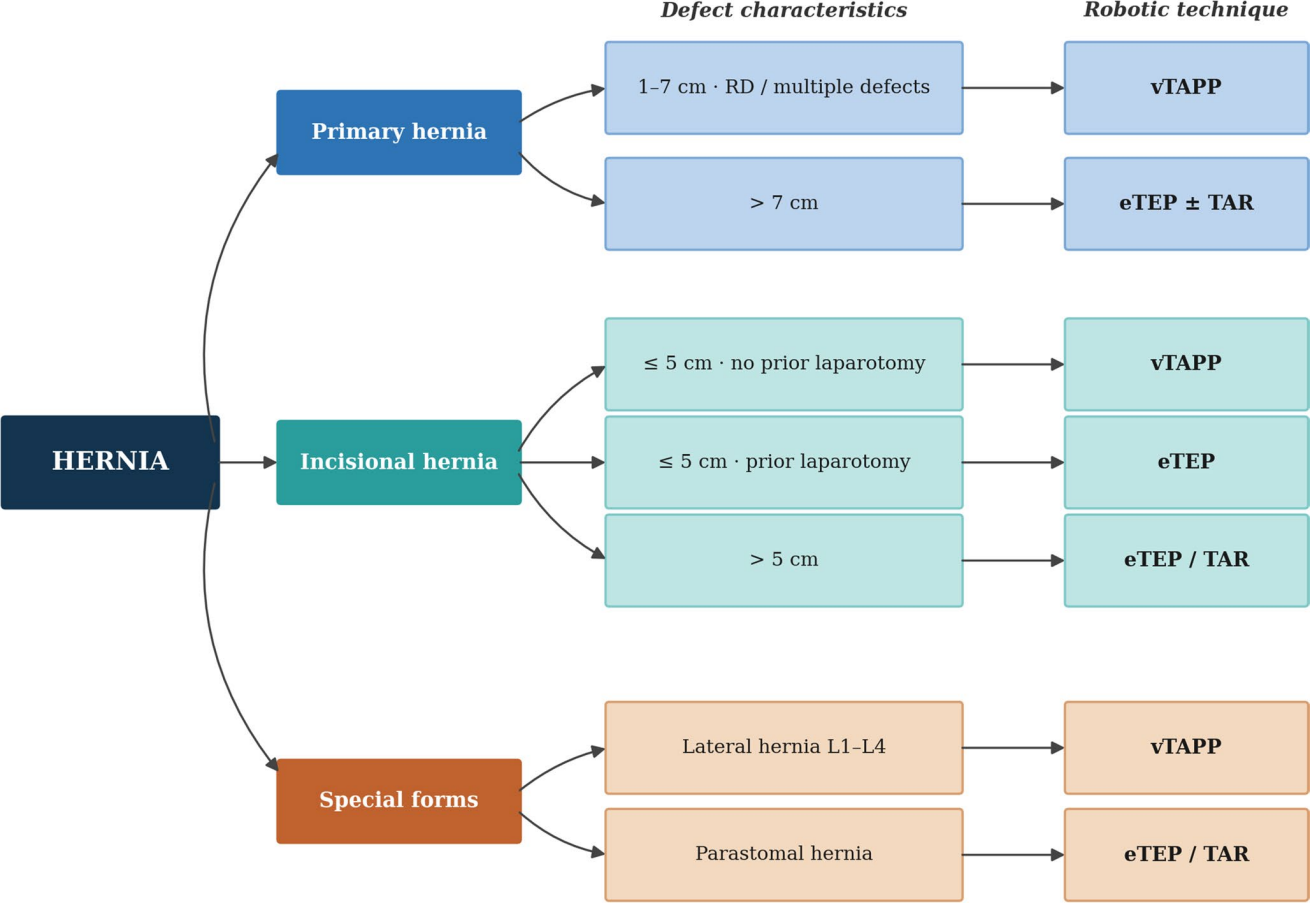
Standardized institutional algorithm for selecting the robotic technique according to hernia category and defect characteristics. RD = rectus diastasis; vTAPP = ventral transabdominal preperitoneal repair; eTEP = enhanced-view totally extraperitoneal; TAR = transversus abdominis release

In the subgroup with Grade II/III obesity (BMI ≥ 35 kg/m², *n* = 60), a notable, albeit non-significant, trend was observed. In the prophylaxis group, a rate of 30.4% (14/46) of 30-day SSOs was observed, whereas this was significantly lower in the control group at 7.1% (1/14) (OR 4.02; 95% CI 0.67–24.22; *p* = 0.155). The wide confidence interval and the small denominator in the non-prophylaxis group do not allow for a causal interpretation; rather, the finding most likely reflects confounding by indication, due to the selective administration of prophylaxis in obese patients with additional risk factors. A similar, though non-significant, trend was also observed among smokers (OR 1.99; 95% CI 0.65–6.05; *p* = 0.278). Patients with diabetes mellitus (*n* = 31) and patients on chronic anticoagulation (*n* = 46) showed no evidence of a differentiated treatment effect. Three further pre-specified subgroup analyses were performed. Stratification by mesh position revealed no association between prophylaxis and 30-day SSO for submuscular/retromuscular repair (eTEP and eTEP/TAR; OR 0.77, 95% CI 0.33–1.82; *p* = 0.65) or preperitoneal repair (vTAPP; OR 2.05, 95% CI 0.54–7.79; *p* = 0.33). An infection-risk composite (diabetes mellitus, age ≥ 75 years, hepatic insufficiency, or significant cardiac comorbidity) likewise showed no significant benefit of prophylaxis in higher-risk patients (OR 1.26, 95% CI 0.45–3.53; *p* = 0.79) or lower-risk patients (OR 1.47, 95% CI 0.53–4.05; *p* = 0.47) (Fig. 2). Finally, stratification by hernia category showed no significant association in either group: primary hernias (*n* = 86) OR 1.88 (95% CI 0.29–12.14; *p* = 0.66) and incisional/recurrent hernias (*n* = 172) OR 1.16 (95% CI 0.53–2.52; *p* = 0.84). As expected, the two categories differed substantially in complexity (primary: median defect 9 cm², operative time 91 min; incisional/recurrent: median defect 40 cm², operative time 131 min), which is why they were analysed as stratified subgroups rather than pooled (Fig. 3).

**Table 4:** Subgroup analyses for 30-day SSO (full cohort)

Subgroup / Stratum	*n*	SSO with AB	SSO no AB	OR (95% CI)	*p*
Hernia category					
Primary	86	5/58 (8.6%)	1/28 (3.6%)	1.88 (0.29–12.14)	0.66
Incisional / recurrent	172	26/116 (22.4%)	11/56 (19.6%)	1.16 (0.53–2.52)	0.84
*Operative technique*					
eTEP	116	16/88 (18.2%)	6/28 (21.4%)	0.79 (0.28–2.19)	0.78
TAPP	98	7/52 (13.5%)	3/46 (6.5%)	2.05 (0.54–7.79)	0.33
eTEP/TAR	44	8/34 (23.5%)	3/10 (30.0%)	0.69 (0.16–3.04)	0.69
*BMI category*					
Normal (< 25)	45	5/31 (16.1%)	1/14 (7.1%)	1.87 (0.27–12.75)	0.65
Overweight (25–30)	79	6/53 (11.3%)	4/26 (15.4%)	0.68 (0.19–2.52)	0.72
Obesity I (30–35)	74	6/44 (13.6%)	6/30 (20.0%)	0.64 (0.19–2.11)	0.53
Obesity II/III (≥ 35)	60	14/46 (30.4%)	1/14 (7.1%)	4.02 (0.67–24.22)	0.16
*Other strata*					
Wound class I (clean)	210	23/140 (16.4%)	7/70 (10.0%)	1.69 (0.70–4.07)	0.30
Wound class II/III	48	8/34 (23.5%)	5/14 (35.7%)	0.55 (0.15–2.04)	0.48
Non-smoker	183	17/128 (13.3%)	7/55 (12.7%)	1.01 (0.40–2.55)	1.00
Active smoker	75	14/46 (30.4%)	5/29 (17.2%)	1.99 (0.65–6.05)	0.28
No anticoagulation	212	26/151 (17.2%)	7/61 (11.5%)	1.53 (0.64–3.67)	0.40
Anticoagulation	46	5/23 (21.7%)	5/23 (21.7%)	1.00 (0.26–3.84)	1.00
Without diabetes	227	22/150 (14.7%)	10/77 (13.0%)	1.13 (0.51–2.48)	0.84
Diabetes mellitus	31	9/24 (37.5%)	2/7 (28.6%)	1.35 (0.25–7.38)	1.00
*Mesh position*					
Submuscular (eTEP/TAR)	160	24/122 (19.7%)	9/38 (23.7%)	0.77 (0.33–1.82)	0.65
Preperitoneal (vTAPP)	98	7/52 (13.5%)	3/46 (6.5%)	2.05 (0.54–7.79)	0.33
*Infection-risk composite*					
High-risk*	77	14/48 (29.2%)	7/29 (24.1%)	1.26 (0.45–3.53)	0.79
Low-risk	181	17/126 (13.5%)	5/55 (9.1%)	1.47 (0.53–4.05)	0.47

OR > 1 indicates higher SSO rate in patients receiving antibiotic prophylaxis (i.e., favors no prophylaxis). All confidence intervals include unity. *eTEP*  enhanced-view totally extraperitoneal, *TAPP*  transabdominal preperitoneal, *TAR*  transversus abdominis release

## Discussion

In this propensity score-matched analysis of 258 patients who underwent rVHR, it was found that the omission of perioperative antibiotic prophylaxis was not associated with an increased rate of SSOs or SSIs at 30 or 90 days, nor with a prolonged hospital stay or higher rehospitalization rates. The point estimates from the matched analysis (SSO 12.8% vs. 15.4%), univariate regression (OR 1.27; 95% CI 0.62–2.60), and the multivariate adjustment (aOR 1.28; 95% CI 0.58–2.82) consistently converged around the null effect. Although these results primarily serve to generate hypotheses and cannot be considered conclusive evidence, they generate the hypothesis that single-shot prophylaxis may not be essential for safe rVHR in this specific robotic, extraperitoneal setting. These results complement the growing body of evidence supporting selective rather than universal prophylaxis in clean elective surgery.

The present study is based on three observations that support our main conclusion. First, the overall SSI rate of approximately 2% at 30 days, and zero SSIs at 90 days, is significantly lower than the historical reference values of 5–15% for open repair of ventral hernias [24, 25]. These positive results highlight the advantages of contemporary rVHR. The implant is positioned extraperitoneally in the retromuscular space, thereby preventing interaction with the intestinal organs. Since eTEP does not involve opening the abdominal cavity, the risk of bacterial contamination is significantly reduced. Precise defect closure using self-fixing suture material (barbed suture) also minimizes the remaining dead space [10, 12]. In a low-risk setting, the absolute risk reduction that can be achieved through prophylaxis, even assuming a generous estimate of efficacy, is marginal. Second, multivariate adjustment did not result in any significant change in the univariate estimators, suggesting that residual confounding by the recorded covariates cannot adequately explain the null effect. Third, the concordance of the estimators from the matched cohort and the overall cohort argues against an artifact caused by limited sampling during matching.

The present results show significant agreement with several recent studies. Tastaldi et al. analyzed the database of the Americas Hernia Society Quality Collaborative and reported SSI rates below 5% in patients undergoing minimally invasive ventral hernia repair, regardless of the prophylaxis regimen [25]. In a systematic review of the Cochrane Database on antibiotic prophylaxis in hernia repair, Sanchez-Manuel et al. determined a relative risk of 0.65 for SSI with prophylaxis in mesh-based repairs. However, the absolute risk reduction in low-risk populations was less than 2%, and the quality of evidence was rated as low [26]. The recently published guideline from the World Society of Emergency Surgery (2023) acknowledges that the evidence base for universal prophylaxis in elective laparoscopic and robot-assisted repair of ventral hernias is of low certainty [7]. To the best of our knowledge, the present study is one of the first to explicitly address the issue of prophylaxis in a cohort consisting solely of rVHR patients, while incorporating a consistent follow-up at 30 and 90 days. Our findings parallel developments in minimally invasive inguinal hernia surgery, where specialist societies now generally advise against routine antibiotic prophylaxis in laparoscopic/endoscopic repair (TEP/TAPP), reserving it for selected higher-risk patients [27, 28]. The common denominator is the combination of a minimally invasive approach with an extraperitoneal mesh position, which is associated with a very low contamination and infection risk. We emphasize, however, that this rationale is strictly confined to this anatomical plane and must not be extrapolated to laparoscopic intraperitoneal onlay mesh (IPOM) or to open repair, where the contamination profile and wound-healing risk differ fundamentally.

The 2018 WHO Global Guidelines for the Prevention of Surgical Site Infection, which recommend antibiotic prophylaxis for procedures involving mesh or other implants, are framed very broadly and do not differentiate by surgical approach or by the anatomical plane of mesh placement [29]. A useful precedent is again minimally invasive inguinal hernia repair (TEP/TAPP), in which specialist societies now generally advise against routine prophylaxis except in selected higher-risk patients [27]. A position that is itself formally at odds with the broad WHO recommendation, yet has become widely accepted. By analogy, the specific combination of a minimally invasive approach with extraperitoneal (retromuscular/preperitoneal) mesh placement may carry a sufficiently low infection risk that the strict WHO recommendation could, in future, be re-evaluated for this particular setting. We stress that this is proposed only as a hypothesis to be tested in an adequately powered confirmatory trial, and not as a basis for present-day deviation from the guideline, which remains the reference standard until such data are available.

The non-significant trend toward higher SSOs in the Grade II/III obesity subgroup receiving prophylaxis (30.4% vs. 7.1%) requires critical interpretation. The wide confidence interval (0.67–24.22) and the very small denominator in the non-prophylaxis arm (*n* = 14) do not allow for a causal conclusion. The most plausible explanation for the observed effects lies in the preferential administration of prophylaxis to obese patients with additional risk factors (more complex hernia findings, longer operative time, higher ASA classes). This results in confounding by indication, which cannot be completely eliminated by propensity matching. This interpretation is consistent with the data from Kudsi et al., who showed that a BMI ≥ 35 kg/m² alone does not serve as a predictor of unfavorable short-term outcomes after rVHR, provided that recorded confounders are balanced [30]. Nevertheless, the obesity subgroup warrants a prospective study, as the absolute event rate in these patients (15/60, 25%) is many times higher than that of the overall cohort.

The validity of the findings is supported by several methodological strengths. The a priori definition of seven biologically plausible matching covariates, the application of a strict caliper (0.1 of the logit), and the achievement of post-match SMDs close to or below 0.10 for all covariates establish a robust comparison group. This study demonstrates that the combination of three analytical approaches (PSM with the McNemar test, univariate OR, and multivariate logistic regression) enables methodological triangulation. The independent verification of all calculations by an external biostatistician using R led to a significant increase in reliability. The nearly complete capture of 90-day outcomes (99.6%) reduces the risk of follow-up bias. Finally, the homogeneity of the surgical technique, performed by a small group of specialized surgeons using a single robotic platform, reduces the variance that posed a problem in earlier multicenter analyses.

However, certain limitations must be taken into account. First, the ambidirectional design, combining a prospectively enrolled intervention cohort with a historical retrospective control group, is susceptible to secular trends and time-period confounding, including potential gradual evolution of surgical technique, perioperative care, and patient selection across the study period. The single-center setting further limits external generalizability and, together with the non-randomized allocation of prophylaxis, precludes definitive causal inference. Second, the allocation of prophylaxis was not randomized. Despite the use of PSM, unmeasured confounders may persist, such as the surgeon’s preference, the subjective assessment of intraoperative contamination, or microbial colonization patterns. Third, the absolute number of events classified as “SSIs” was very low (five events at 30 days, zero at 90 days). This has significant implications for the confidence intervals and limits the statistical power to detect moderate differences specific to SSIs. A power calculation shows that detecting a 50% relative reduction in SSIs (from 2% to 1%) with 80% power would require approximately 4,400 patients, a sample size that exceeds the scope of single-center observational studies. Fourth, the wound class was retrospectively determined in 14% of cases and was not documented at the time of initial assessment. Fifth, post-discharge follow-up was based on a combination of clinic visits, telephone calls, and review of the electronic medical record. The hypothesis posited in this study is that discrete SSOs treated exclusively on an outpatient basis were not captured. A sixth issue is the inadequate recording of the time interval between the start of antibiotic therapy and the time of skin incision. Consequently, it can be inferred that patients may have received a suboptimally timed prophylaxis. This could result in a bias toward the null effect. Several further limitations should be acknowledged. Antibiotic-associated sequelae (Clostridioides difficile infection, antibiotic-associated diarrhoea), with a reported postoperative incidence of approximately 0.2-8% in surgical patients [31], and allergic/anaphylactic reactions were not captured in the retrospective control cohort and could not be analysed. Pharmacological immunosuppression (e.g., corticosteroids, biologics, chemotherapy) was not recorded discretely, so our infection-risk composite is a pragmatic proxy rather than a validated, standardized SSI risk score, and no standardized risk-assessment instrument (e.g., NNIS/ACS-NSQIP) was available in the retrospective arm. Primary and incisional/recurrent hernias differ materially in operative complexity; we therefore did not pool them but analysed them both as a balanced matching covariate and as a separate stratified subgroup, in which the absence of a prophylaxis effect was consistent across categories. Most importantly, the cohort comprises exclusively robotic extraperitoneal repairs; the findings therefore cannot be generalized to open or laparoscopic repair, in which wound exposure, mesh plane and contamination differ materially [32–34].

Despite these limitations, the results have immediate clinical and health policy implications. Routine universal prophylaxis for rVHR ties up resources, exposes patients to the documented side effects of antibiotics (including microbiome disturbances and rare allergic reactions), and increases selection pressure for resistant pathogens. We note that antimicrobial resistance is driven more by prolonged therapy than by single-dose prophylaxis [14], and that perioperative cephalosporins carry a small but real risk of allergic and, rarely, anaphylactic reactions (perioperative anaphylaxis to cefazolin approximately 0.02%) [35]; neither Clostridioides difficile infection/antibiotic-associated diarrhoea nor hypersensitivity events were captured in our dataset, both of which are acknowledged above. In the context of the current literature, the available data suggest that prophylaxis should be reserved primarily for high-risk patients, such as those with significant immunosuppression, extremely large defects indicating TAR, or specific colonization profiles, rather than being used across the board. The implementation of such a restrictive protocol should be accompanied by prospective monitoring of SSI rates using a non-inferiority margin, so that universal prophylaxis can be reinstated in a timely manner in the event of a clinically significant increase.

This development opens up new perspectives for future research projects. It is recommended to conduct a pragmatic, randomized non-inferiority trial comparing the effects of selective and universal prophylaxis in elective rVHR. Based on the observed incidences, an enrollment of approximately 800 patients per study arm would be statistically sufficient to rule out a 3-percentage-point increase in SSOs with 80% probability. Second, translational studies of the wound microbiome in retromuscular versus intraperitoneal mesh placement could clarify whether prophylaxis acts primarily by reducing the bacterial inoculum or via alternative mechanisms. Third, dedicated subgroup analyses in high-BMI cohorts - ideally through stratified randomization - should address the residual uncertainty remaining in our cohort (obesity grade II/III).

## Conclusion

In a propensity-score-matched analysis of consecutive robot-assisted ventral hernia repairs, the omission of perioperative antibiotic prophylaxis was not associated with a significant increase in SSO, SSI, or LOS at 30 or 90 days. The convergence of matched, univariate, and multivariable estimates around the null, combined with an observed SSI rate of approximately 2%, is consistent with non-inferiority of a no-prophylaxis strategy. Formal demonstration of non-inferiority in a randomized controlled trial, assuming an 18% baseline SSO rate, a non-inferiority margin of 5% points, two-sided α = 0.05, and 80% power, would require approximately 930 patients per arm (> 1,800 in total), a sample size that is not achievable within a single-centre setting and has not been reached in any published multicentre hernia trial to date. Taken together, these findings support the hypothesis that routine single-shot prophylaxis may not be essential in this specific robotic, extraperitoneal setting, in which the contamination risk is inherently low. They are hypothesis-generating and define the scope of the question rather than a clinical recommendation: they apply to the minimal-invasiv, extraperitoneal approach and should not be extrapolated to laparoscopic intraperitoneal onlay mesh (IPOM) or to open repair, where the contamination profile and wound-healing risk differ fundamentally, while current guideline-recommended practice - including the WHO Global Guidelines for the Prevention of Surgical Site Infection - remains the reference standard until confirmatory data are available. A selective, risk-adapted prophylaxis strategy is therefore a clinically meaningful and testable hypothesis, which an adequately powered prospective randomized non-inferiority trial should now address.

## Data Availability

De-identified data and analytic code are available from the corresponding author upon reasonable request, subject to institutional approval.
